# Myocardial Infarction Can Be Safely Excluded by High‐sensitivity Troponin I Testing 3 Hours After Emergency Department Presentation

**DOI:** 10.1111/acem.13922

**Published:** 2020-03-27

**Authors:** W. Frank Peacock, Robert Christenson, Deborah B. Diercks, Christian Fromm, Gary F. Headden, Christopher J. Hogan, Erik B. Kulstad, Frank LoVecchio, Richard M. Nowak, Jon W. Schrock, Adam J. Singer, Alan B. Storrow, Joely Straseski, Alan H. B. Wu, Daniel P. Zelinski

**Affiliations:** ^1^ Emergency Medicine Baylor College of Medicine Houston TX; ^2^ Department of Pathology University of Maryland Baltimore MD; ^3^ Department of Emergency Medicine UT Southwestern Medical Center Dallas TX; ^4^ Department of Emergency Medicine Einstein Healthcare Network Philadelphia PA; ^5^ Department of Emergency Medicine Medical University of South Carolina Charleston SC; ^6^ Department of Emergency Medicine Virginia Commonwealth University Richmond VA; ^7^ Department of Emergency Medicine Banner Health Phoenix AZ; ^8^ Department of Emergency Medicine Henry Ford Health System Detroit MI; ^9^ Department of Emergency Medicine Case Western University Cleveland OH; ^10^ Department of Emergency Medicine Stonybrook University Stonybrook NY; ^11^ Department of Emergency Medicine Vanderbilt University Medical Center Nashville TN; ^12^ ARUP Laboratories University of Utah School of Medicine Salt Lake City UT; ^13^ Department of Pathology University of California San Francisco CA; ^14^ Emergency Department Dublin Methodist Hospital Dublin OH

## Abstract

**Background:**

The accuracy and speed by which acute myocardial infarction (AMI) is excluded are an important determinant of emergency department (ED) length of stay and resource utilization. While high‐sensitivity troponin I (hsTnI) >99th percentile (upper reference level [URL]) represents a “rule‐in” cutpoint, our purpose was to evaluate the ability of the Beckman Coulter hsTnI assay, using various level‐of‐quantification (LoQ) cutpoints, to rule out AMI within 3 hours of ED presentation in suspected acute coronary syndrome (ACS) patients.

**Methods:**

This multicenter evaluation enrolled adults with >5 minutes of ACS symptoms and an electrocardiogram obtained per standard care. Exclusions were ST‐segment elevation or chronic hemodialysis. After informed consent was obtained, blood samples were collected in heparin at ED admission (baseline), ≥1 to 3, ≥3 to 6, and ≥6 to 9 hours postadmission. Samples were processed and stored at –20°C within 1 hour and were tested at three independent clinical laboratories on an immunoassay system (DxI 800, Beckman Coulter). Analytic cutpoints were the URL of 17.9 ng/L and two LoQ cutpoints, defined as the 10 and 20% coefficient of variation (5.6 and 2.3 ng/L, respectively). A criterion standard MI diagnosis was adjudicated by an independent endpoint committee, blinded to hsTnI, and using the universal definition of MI.

**Results:**

Of 1,049 patients meeting the entry criteria, and with baseline and 1‐ to 3‐hour hsTnI results, 117 (11.2%) had an adjudicated final diagnosis of AMI. AMI patients were typically older, with more cardiovascular risk factors. Median (IQR) presentation time was 4 (1.6–16.0) hours after symptom onset, although AMI patients presented ~0.5 hour earlier than non‐AMI. Enrollment and first blood draw occurred at a mean of ~1 hour after arrival. To evaluate the assay's rule‐out performance, patients with any hsTnI > URL were considered high risk and were excluded. The remaining population (*n* = 829) was divided into four LoQ relative categories: both hsTnI < LoQ (Lo‐Lo cohort); first hsTnI < LoQ and 2nd > LoQ (Lo‐Hi cohort); first > LoQ and second < LoQ (Hi‐Lo cohort); or both > LoQ (Hi‐Hi cohort). In patients with any hsTnI result <20% CV LoQ (Groups 1–3), *n* = 231 (23.9% ruled out), AMI negative predictive value (NPV) was 100% (95% confidence interval [CI] = 98.9% to 100%). In patients with any hsTnI below the 10% LoQ, *n* = 611 (58% rule out), AMI NPV was 100% (95% CI = 99.5% to 100%). Of the Hi‐Hi cohort (i.e., no hsTnI below the 10% LoQ, but both < URL), there were four AMI patients, NPV was 98.2% (95% CI = 95.4% to 99.3%), and sensitivity was 96.6.

**Conclusions:**

Patients presenting >3 hours after the onset of suspected ACS symptoms, with at least two Beckman Coulter Access hsTnI < URL and at least one of which is below either the 10 or the 20% LoQ, had a 100% NPV for AMI. Two hsTnI values 1 to 3 hours apart with both < URL, but also >LoQ had inadequate sensitivity and NPV.

Of the more than 145 million annual emergency department (ED) visits, it is estimated that 7.6 million patients present with a chief complaint of chest pain.^1^ An additional 3.4 million and 2.8 million will have complaints of the potential anginal equivalents of shortness of breath or vomiting, respectively. Because manifestations of coronary artery disease represent the number one cause of death in the United States, and the incidence rate of acute myocardial infarction (AMI) in these populations is often less than 20%, the initial evaluation in these nearly 14 million patients is intended to safely exclude the diagnosis of AMI. This is necessary because patients discharged from the ED with an undiagnosed AMI have worse outcomes that may include mortality. No contemporary estimates of the missed MI rate exist, but it is a generally unacceptable outcome, the concern for which results in extensive (and most commonly negative) evaluations. Although no MI recognition strategy has demonstrated infallibility, surveys of emergency physicians suggest an acceptable AMI and subsequent multiple adverse cardiac event miss rate not exceeding 1% is desirable.^2^ Historically, successful strategies demonstrating a negative predictive value (NPV) in excess of 99% required biomarker testing for at least 6 hours after presentation, which were then followed by further risk stratification techniques (e.g., myocardial perfusion evaluations).

High‐sensitivity troponin assays have held the promise of a more accurate and rapid AMI rule outs. Unfortunately, due to a challenging regulatory environment, the United States is a decade behind the rest of the world in troponin research and clinical availability. It is only in the past 2 years that any high‐sensitivity troponin assay was FDA cleared and thus available for U.S. physicians. This has resulted in the majority of the high‐sensitivity troponin literature originating from European populations that have markedly higher AMI rule in rates than in the United States. Since the rates of MI are much lower in the United States, our different pretest odds create challenges in understanding the predictive values of high‐sensitivity troponin when applied to our population.

The availability of high‐sensitivity troponin assays has created an opportunity to shorten the time required for evaluation, as well as increase the number of patients who may be safely discharged based on the reported values, or changes in reported values, undetectable by contemporary assays. However, to minimize the probability of patient's misclassification,^3^ the ability to measure low concentrations of an analyte must be considered in conjunction with the precision of that result, as it is not useful to have sensitive but imprecise troponin assays. The analytic imprecision of an assay, termed the coefficient of variation (CV), generally increases with decreasing analyte concentrations, and the level of quantification (LoQ) is the cutpoint at which a predefined CV is observed. The FDA requires a CV of ≤20% for the reporting of clinical results; however, the CV of ≤10% has been suggested as optimal at the decision point.^3^, ^4^ While high‐sensitivity troponin I (hsTnI) levels above the 99th percentile (defined as the upper reference level [URL]) are used to define high risk, the absence of high risk does not equate to a rule out, and lower levels are required to safely exclude a subsequent diagnosis of AMI. Few emergency medicine studies have presented data using different LoQs as a rule‐out cutpoint, so the consequence on clinical impact is unclear.^4^, ^5^ Since LoQ values are highly assay‐specific, our objective was to evaluate the Beckman Coulter hsTnI assay using LoQ cutpoints of 10 and 20% CV as rule‐out cutpoints, to exclude MI within 3 hours of ED presentation in patients with suspected acute coronary syndromes (ACSs).

## Methods

The database used for this analysis was prospectively collected during 2012 and 2013 as a part of an FDA submission for the Beckman Coulter hsTnI assay, with a patient cohort used in the evaluation of the AccuTnI+3 contemporary TnI assay and is described elsewhere.^7^ Local data are kept by the enrolling institution a minimum of 7 years after the FDA clearance and are kept in conglomerate by both the sponsor and the FDA. The analysis for this study was performed by Beckman Coulter.

The original study was a convenience sample investigation enrolling adult patients (age ≥21 years) who presented to 14 geographically diverse, hospital‐associated EDs. All participating hospitals obtained institutional review board approval and reflected urban, suburban, and rural patient populations. Eligible patients had an electrocardiogram (ECG) obtained as a part of their standard of care and reported at least 5 minutes of symptoms consistent with ACSs that included chest pain, shortness of breath, left arm pain, lightheadedness, dizziness, weakness, or syncope. Patients were excluded if the initial ECG demonstrated ST‐segment elevation MI (STEMI), if they were on chronic hemodialysis, or if they were unable to provide informed consent. The primary endpoint was an adjudicated diagnosis of AMI.

After informed consent was obtained, serial blood samples were collected in lithium heparin tubes per the local standard of care and grouped into four different time frames: time of admission (baseline), ≥1 to 3, ≥3 to 6, and ≥6 to 9 hours after baseline. Samples were centrifuged and then stored at –20°C within 1 hour of blood draw (the timing of which was reported in the FDA submission to not have analytic consequence). Plasma aliquots were tested at three independent clinical laboratories on the Access hsTnI DxI 800 immunoassay system (Beckman Coulter). The original study enrolled 1,929 patients of whom 75 were excluded due to insufficient sample volume and was powered for detection of 73 to 139 MIs with 90% to 95% diagnostic sensitivity.^7^ An additional 805 patients were excluded from the present retrospective analysis due to a missing baseline or 3‐hour blood draw (these patients included those that developed a STEMI or were discharged/left the ED after a single sample).

The Beckman Coulter Access hsTnI assay is a two‐site immunoenzymatic (“sandwich”) assay. For this assay, monoclonal anti–cTnI antibody conjugated to alkaline phosphatase is added to a reaction vessel along with a surfactant containing buffer and sample. After a short incubation period, paramagnetic particles coated with monoclonal anti–cTnI antibody are added. The sample cTnI then binds to the anti–cTnI antibody on the solid phase, while the anti–cTnI antibody–alkaline phosphatase conjugate reacts with a different antigenic site on the cTnI molecule. Materials bound to the solid phase are held in a magnetic field while unbound materials are washed away. Chemiluminescent substrate is added to the vessel, and light generated by the reaction is measured with a luminometer. The light production is directly proportional to the concentration of cTnI in the sample. The amount of analyte in the sample is determined from a stored, multipoint calibration curve, the analytical performance of which has been previously described.^8^, ^9^, ^10^, ^11^, ^12^, ^13^, ^14^, ^15^

### Data Analysis

For this analysis, the URL for both sexes was defined as 17.9 ng/L and was derived from data that served as part of the FDA submission for this assay, as is documented in the package insert.^7^, ^16^ The appropriate sample size was a priori determined for the FDA submission, but not for this secondary analysis. The LoQ was defined as the 20% CV cutpoint, occurring at 2.3 ng/L TnI, and a higher 10% CV cutpoint, which equaled 5.6 ng/L TnI. A clinically significant delta (that exceeding the larger of the CV's used in this analysis) was subsequently defined as an hsTnI change >25%.

A clinical criterion standard index MI diagnosis was adjudicated by an independent clinical endpoints committee that consisted of four cardiologists, using criteria consistent with the Third Universal Definition of MI and the local troponin.^4^ Adjudicators were blinded to the Beckman Coulter assay results. Because the intent of this analysis was to identify low‐risk cutpoints, patients adjudicated as MI were not differentiated into subtypes. All results presented here were based on the adjudicated diagnoses using the local contemporary troponin assay.^7^ The overall patient population was characterized using descriptive statistics. Outcome rates are presented with sensitivity, specificity, and predictive values.

To evaluate the rule out capability of the assay, any patient with either a time 0 or a 1‐to 3‐hour hsTnI above the URL was considered higher risk and was excluded from the AMI rule‐out category. The remaining population with TnI values below the URL at both time points could fall into one of four prospectively defined categories based on hsTnI levels relative to the two selected LoQ's. They could have both measures below the LoQ (Lo‐Lo cohort), the first measure below and the second above the LoQ (Lo‐Hi cohort), the first above and the second below the LoQ (Hi‐Lo cohort), or both concentrations could be above the LoQ (Hi‐Hi cohort).

## Results

Overall, 1,049 patients met the entry criteria of serial hsTnI results available at baseline and between 1 and 3 hours. Demographic data using descriptive statistics and measures of dispersion are presented in Table 1. A total of 117 (11.2%) patients had an adjudicated diagnosis of MI. Those adjudicated as having a MI were older and more likely male with prior coronary artery disease and with a history of revascularization procedures as well as having known cardiovascular risk factors of hypertension, diabetes, tobacco use, heart failure, and renal disease. Patients presented a median (interquartile range [IQR]) of 4 (1.6–16.0) hours after symptom onset, although those diagnosed with MI presented almost 0.5 hour earlier. Enrollment and first blood draw occurred approximately 1 hour after arrival. Compared to the excluded patients (Table 1), the patients in this study were slightly younger and there were more females, with rates of prior coronary procedures, and they experienced a longer wait from presentation to first blood draw, with most of the significant differences occurring in non‐MI group.

**Table 1:** Demographics and Comparison of Analytic (*n* = 1,049) and Excluded Subgroups (*n* = 805)

Category	Subgroup (*n* = 1,049)		Not in Subgroup (*n* = 805)		p‐value	
	Non‐MI, *n* = 932 (88.8%)	MI, *n* = 117 (11.2%)	Non‐MI, *n* = 684 (85.0%)	MI, *n* = 121 (15.0%)		
					Non‐MI	MI
Age (years), median (IQR)	55 (48‐64)	62 (53‐72)	56 (49‐68)	62 (51‐72)	0.0002	0.7744
Age ≥ 60 years	36.1%	55.6%	41.5%	57.0%	0.0255	0.8197
Male	48.5%	66.7%	57.9%	73.6%	0.0002	0.2466
From symptom onset to presentation	4.2 (1.6‐16.8)	3.7 (1.5‐12.5)	3.9 (1.7‐16.0)	2.9 (1.3‐14.2)	0.1124	0.1769
From presentation to the first blood draw	1.4 (1.0‐1.9)	1.2 (1.0‐1.8)	1.0 (0.5‐1.5)	0.7 (0.4‐1.1)	<0.0001	<0.0001
Asian	1.0%		3.4%	4.1%	0.2631	0.4324
African American	39.1%	34.2%	23.8%	23.1%		
White	55.5%	58.1%	63.7%	59.5%		
Hypertension	70.3%	76.9%	69.0%	77.7%	0.5765	0.9505
Hypercholesterolemia	51.1%	58.1%	55.3%	55.4%	0.0652	0.8105
Diabetes mellitus	29.3%	35.0%	28.4%	33.1%	0.7027	0.7471
Current smoker	29.8%	24.8%	23.7%	33.1%	0.0110	0.1224
Past smoker	30.4%	41.9%	34.6%	38.0%	0.0317	0.7220
Known >50% coronary stenosis	27.5%	46.2%	30.4%	43.8%	0.0494	0.9876
MI	21.2%	35.9%	24.1%	32.2%	0.0973	0.7428
Coronary stent or angioplasty	18.7%	31.6%	26.5%	36.4%	<0.0001	0.4027
CABG	9.0%	14.5%	13.2%	14.9%	0.0067	0.9400
Heart failure	16.0%	28.2%	13.5%	20.7%	0.2960	0.2012
CKD	9.3%	13.7%	6.9%	13.2%	0.1140	0.9188

CABG = coronary artery bypass graft; CKD = chronic kidney disease; IQR = interquartile rank; MI = myocardial infarction.

**Figure 1:**
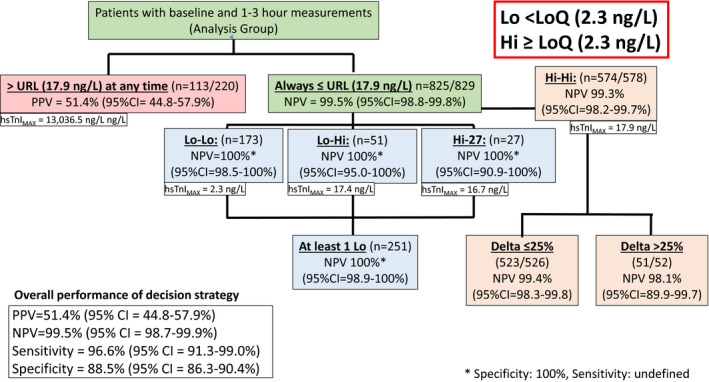
Distribution of patients with all hsTnI below the upper reference level (17.9 ng/L), and serial hsTnI as stratified by above (Hi) or below (Lo) the 20% LoQ (2.3 ng/L). URL= upper reference level, defined as the 99th percentile of a normal population. LoQ = level of quantification. PPV = positive predictive value. NPV = negative predictive value. hsTnI = high sensitive troponin I. hsTnImax = the highest hsTnI level in the cohort. 95% CI = 95% confidence interval. Delta = hsTnI difference between 1st and 2nd levels. All hsTnI were below the URL for this analysis. Lo‐Lo = both hsTnI levels below the LoQ. Lo‐Hi = 1st hsTnI < LoQ, 2nd > LoQ. Hi‐Lo = 1st hsTnI > LoQ, 2nd < LoQ. Hi‐Hi = both hsTnI >LoQ.

**Figure 2:**
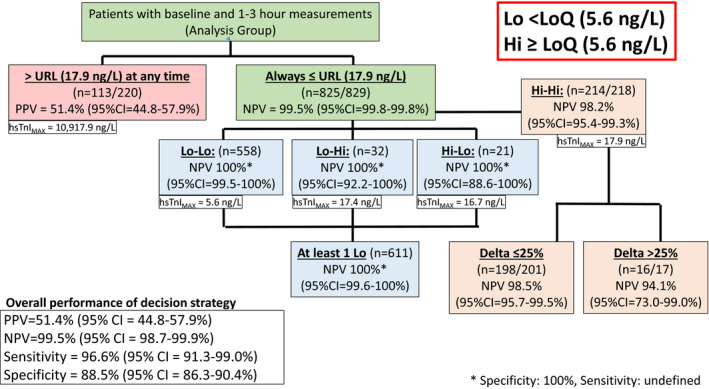
Distribution of patients with all hsTnI below the upper reference level (17.9 ng/L), and serial hsTnI as stratified by above (Hi) or below (Lo) the 10% LoQ (5.6 ng/L). URL= upper reference level, defined as the 99th percentile of a normal population. LoQ = level of quantification. PPV = positive predictive value. NPV = negative predictive value. hsTnI = high sensitive troponin I. hsTnImax = the highest hsTnI level in the cohort. 95% CI = 95% confidence interval. Delta = hsTnI difference between 1st and 2nd levels. All hsTnI were below the URL for this analysis. Lo‐Lo = both hsTnI levels below the LoQ. Lo‐Hi = 1st hsTnI < LoQ, 2nd > LoQ. Hi‐Lo = 1st hsTnI > LoQ, 2nd < LoQ. Hi‐Hi = both hsTnI >LoQ.

Of the total 1,049 patients, 220 had at least one hsTnI value above URL, of whom 113 were diagnosed with MI. Four additional AMI's were adjudicated among 829 patients with both hsTnI values below the URL (see Figures 1 and 2). The diagnostic accuracy of using the URL cutpoint was as follows: sensitivity = 96.6% (95% CI = 91.3% to 99.0%), specificity = 88.5% (95% CI = 86.3% to 90.4%), positive predictive value (PPV) = 51.4% (95% CI = 44.8% to 57.9%), and NPV = 99.5% (95% CI = 98.7% to 99.9%). The high NPV obtained with this strategy did result in a poor PPV (51.4%) such that the clinical utility of a positive troponin in this setting is as likely as not to represent a MI.

The analysis of lower‐risk cohorts is also shown in Figures 1 and 2. For either value of the LoQ, none of the patients with at least one hsTnI reading below LoQ (Lo‐Lo, Lo‐Hi, Hi‐Lo) were diagnosed with MI. Using the 20% CV LoQ, this strategy would identify 251 patients (24% of the total population) in whom the diagnosis of MI could be excluded with NPV = 100% (95% CI = 98.7% to 100.0%). The majority of patients in this cohort had both measures below the LoQ (*n* = 173, 69%), with fewer having a rising pattern with the second measure of hsTnI above the LoQ (*n* = 51, 20%). The fewest number had a falling hsTnI pattern (*n* = 27, 11%), with the first hsTnI above, and the second below, the LoQ. Of 51 subjects with a rising hsTnI pattern, the median (IQR) change was 0.7 (0.4–1.1 ng/L), with a maximum value of 16.7 ng/L. Performing the same analysis, with the 10% CV LoQ, resulted in an increase in the number of patients ultimately ruled out for MI to 611 (58% of the total population), while still maintaining a 100% NPV (95% CI = 99.6% to 100%) in the combined Lo‐Lo, Lo‐Hi, and Hi‐Lo groups.

Four patients in Hi‐Hi group with both hsTnI values between either LOQ (10% or 20%) and the URL, were adjudicated as MI (NPVs = 99.3 and 98.2%, respectively). Of these four, three had a local standard‐of‐care TnI above the local TnI URL within 3 hours, and the fourth had a standard of care TnI elevation identified 12 hours after presentation. In contrast, three high‐risk (any hsTnI > URL) patients had an initial hsTnI below the LoQ and a second troponin above URL, but were ultimately adjudicated as non‐MI. We also sought to determine if the magnitude of temporal fluctuations in troponin concentration in the Hi‐Hi cohort (TnI < URL but >LoQ) could provide diagnostically useful information. Only four subjects in this subpopulation experienced an MI, making any conclusion from such limited data impractical.

An important question is if a single hsTnI < LoQ at baseline is sufficient for an AMI rule out. The sensitivity, specificity, PPVs, and NPVs for the 20% LoQ (*n* = 224) were 100, 24, 14.2, and 100%, respectively, and 100, 63, 25.7, and 100%, for the 10% LoQ (*n* = 590), respectively. The fact that this approach was effective must be considered by the fact that the number of patients with early presentations is limited, and the clinician must precisely know the time of symptom onset (a challenging feat in some patients). The advantage of a two‐blood‐draw strategy is that it ensures that all patients have at least 3 hours of symptoms, or their symptoms have resolved for at least 3 hours.

Delineation of the cohort who presented with a baseline hsTnI < LoQ and subsequently ruled in for AMI (the penalty for not waiting for the second hsTnI) showed that, of the total population, 224 had a baseline hsTnI below the 20% LoQ, of whom zero subsequently ruled in for AMI. If the baseline hsTnI was below the 10% LoQ, then 593 patients were included, of whom three subsequently ruled in for AMI after 3 hours. Statistical performance of hsTnI in the early presenters provided a sensitivity, specificity, PPV, and NPV of 100, 26, 14.7, and 100% and 100, 65.6, 26.7, and 1000% for the 20% LoQ and the 10% LoQ, respectively. Specificity and PPV using the LoQ cutpoint alone are very low. However, a value below the LoQ is suggested as a ‘rule‐out’ criteria, not a ‘rule‐in’ criteria. Rule‐in criteria should utilize the URL in line with current guidelines. This will provide much higher specificity for index MI.

**Figure 3:**
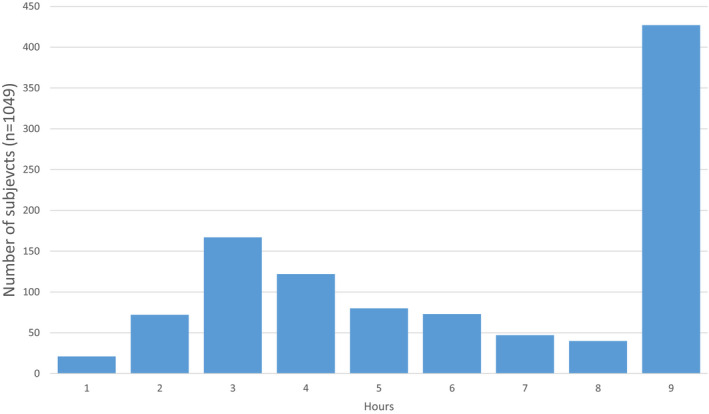
Time from symptom onset to first blood draw

The distribution of the time from symptom onset to blood draw is presented in Figure 3. Note that ~30% of patients presented in the first 3 hours after symptom onset. A sensitivity analysis was performed in the 260 patients who presented to the ED within 3 hours of symptom onset. The incidence of MI in this “early presenter” population was 12.7% (33/260), and the NPV of both hsTnI values <URL was 99.5% (95% CI = 97.3% to 99.9%) for both LoQ values. This was similar to that of patients presenting after 3 hours of symptom onset, regardless of the LoQ cutpoint used, NPV = 99.5% (95%CI = 97.3% to 99.9%) for both LoQ values (see Table 2). While the sensitivity of the approach was 97.0% (95% CI = 83.0% to 100.0%), which is similar to that of the overall analysis, the low number of patients, and resulting wide CIs preclude reasonable extension of this finding to clinical practice.

**Table 2:** Early Presenters Stratified by LoQ and Rule Out or Non‐Rule Out Status

LoQ (ng/L)	Non–rule‐out Group (>17.9 ng/L at Baseline and/or 1–3 Hours)	Rule‐out Group (≤17.9 ng/L at Baseline and 1–3 Hours)
2.3	MI = 32, PPV = 61.5% (32/52) (95% CI = 48.0%–73.5%) Specificity = 91.2% (207/227) (95% CI = 48.0%–73.5%)	MI = 1, NPV = 99.5% (207/208) (95% CI = 97.3%–99.9%) Sensitivity = 97.0% (32/33) (95% CI = 83.0%‐100.0%)
5.6	MI = 32, PPV = 61.5% (32/52) (95% CI = 48.0%–73.5%)	MI = 1, NPV = 99.5% (207/208) (95% CI = 97.3%–99.9%)

LoQ = level of quantification; MI = myocardial infarction; NPV = negative predictive value; PPV = positive predictive value.

## Discussion

In this prospective evaluation of an all comers ED population of patients presenting with potential acute coronary symptoms, we found that no patient with two hsTnI measurements below the URL cutpoint, and with at least one of which was below either the 10% or the 20% LoQ, had a diagnosis of AMI (Figures 1 and 2). It is important to note that being below the URL “rule‐in cutpoint” does not equate to a rule out. Four patients with both levels below the URL were adjudicated as AMI and the sensitivity of this strategy was only 96.6%. However, considering the LoQ as a “rule‐out” cutpoint, if one or both of the two values is below this LoQ, it represents a highly effective strategy for expediting management decisions in ED patients because it has a sensitivity and NPV of 100%. While other studies have utilized similar strategies,^17^ we are not aware of any that have reported NPVs of 100% for AMI in an “all‐comers” ED patient population, while still ruling out up to 58% of the presenting population.

Our examination of two different LoQ values demonstrated that use of the higher 5.6 ng/L decision point, corresponding to a 10% CV LoQ, did increase the number of AMI rule outs, from 23.9% to 58% of all patients meeting the inclusion criteria, while still maintaining a 100% NPV. The high NPV obtained with LoQ‐based strategy did result in a poor PPV (26.7%, 95% CI = 22.8% to 31.1%) requiring further risk stratification through clinical gestalt or risk‐scoring approaches,^13^ particularly in patients with both hsTnI values falling between LoQ and URL. Clinicians should still consider that hsTnI values on the upper end of the normal range have been associated with adverse long‐term patient outcomes.^13^, ^18^ In fact, in this study four patients within Hi‐Hi cohort were diagnosed with MI. While earlier international publications on using Access hsTnI for rapid rule‐out algorithms allowed only the consideration of the baseline hsTnI,^15^ our suggested approach is fully consistent with the fourth universal definition of MI and current U.S. guidelines for NSTEMI,^19^ because it relies on the sequential draws and considers patients with at least one value above URL as high risk.

Additionally, higher hsTnI values above the LoQ and below the URL were clinically useful, but only if the lower cutpoint was applied (LoQ = 20%, hsTnI = 2.3 ng/L). In this scenario, the lack of a detectable hsTnI increase (defined as delta <25% from baseline) had a NPV of 99.4% (95% CI = 98.3% to 99.9%) and was adequate for clinical decision making. However, if the physician used the higher LoQ (e.g., 5.6 ng/L), or the patient’ hsTnI was above 25%, the acceptable clinical threshold of an NPV above 99% is not met.

We addressed the challenge of defining a relevant troponin change as that exceeding 25%. We chose a 25% delta as the smallest possible relevant change to exceed the 20% CV, because a delta below the CV may simply represent laboratory error without clinical consequence. A percent delta is a somewhat arbitrary decision as we could have chosen absolute changes. The challenge of defining a percent delta is that when troponin levels are low, a percent change may represent clinically irrelevant changes (e.g., a change from 3 to 4 ng/L represents a 25% delta, but is only 1 ng/L). Conversely, we could have selected an absolute troponin change. This strategy is superior for the identification of low‐level changes, but when applied to elevated troponins may not reflect a clinically relevant change. For example, a change of 5 ng/L in a patient with an initial hsTnI of 5 ng/L represents a doubling of the result and is likely to be clinically relevant, while a change of 5 ng/L in a patient with a troponin of 60 ng/L is likely not correlated with differences in outcomes or therapy.

It should be pointed out that certain cohorts of patients were excluded from this study and thus should be excluded from clinical adoption of an early rule‐out strategy with this assay. This included patients with STEMI on initial ECG and those on dialysis. Further, because of the mechanics of performing a study (i.e., obtaining informed consent), the mean time for blood draw was 1 hour after ED arrival. While this represents earlier sampling than other previously published high‐sensitivity troponin studies evaluating AMI rule out^17^ and is consistent with most ED patients presenting with the onset of ACS symptoms relative to timing of hsTnI measurements, the consideration of symptom onset is critical in diagnostic decision making. Patients presenting in less than 4 hours after symptom onset should not be considered to have ruled out with the strategy described herein until they have been able to have hsTnI measurements obtained at baseline and 3 hours afterward.

Many studies have been published using an accelerated diagnostic protocol strategy in the management of ED suspected ACS patients.^20^, ^21^, ^22^, ^23^ These generally use risks scores (e.g., EDACS, HEART), ECG, and serial troponin obtained at baseline and 1, 2, or 3 hours. What is clear is that, as the sensitivity of the clinically available assays have improved, so have safe ED discharge rates as evaluated by 30‐day outcomes. We cannot comment on safe discharge rates, since 30‐day outcomes were not part of this study. Nonetheless, our findings strongly support the utilization of a high‐sensitivity troponin assay for excluding AMI diagnosis in an ED setting.

## Limitations

This analysis evaluates the diagnostic value for AMI of the Beckman Coulter high‐sensitivity troponin assay in a large prospectively obtained sample set. Post‐ED discharge prognostic claims cannot be considered, because only the incident visit was evaluated by the physicians performing the adjudication. Further, since we only evaluated for the diagnosis of AMI and did not consider clinical risk scoring, myocardial perfusion evaluation (e.g., stress testing), or MI type, ED disposition should be considered with the possible necessity of further downstream testing. Additionally, this study was based on the gathering of samples for diagnosis but did not alter the standard of care in real time, such that future prospective studies may be needed to evaluate 30‐day outcomes as a result of acting on this assay's information in real time. Also, the present analysis and observations were based on a retrospective data exploration of a prospectively collected population. Consequently, some cohorts were of limited size and had larger CIs that should be considered if applying these results to patients with matching characteristics. Moreover, the percentage of patients with an index MI is small (typical for U.S. populations) and this makes the NPV particularly high compared to sensitivity. These factors further underscore the need for prospective studies with prespecified endpoints and adequate statistical power to confirm our findings. Also, while some have supported the use of sex‐specific cutpoints for the diagnosis of AMI, in regard to this assay, proof of the consequence of applying sex‐specific cutpoints^12^, ^13^, ^14^ awaits the completion of additional investigations. Finally, patients presented a median of 3 hours after the onset of symptoms and had blood collected for this study an hour later, so that outcomes in individuals presenting earlier warrant further investigation.

## Conclusions

Patients presenting >3 hours after the onset of suspected acute coronary syndrome symptoms, with at least two Beckman Coulter Access high‐sensitivity troponin I < upper reference level, and at least one of which is below either the 10% or 20% level of quantification, had a 100% negative predictive value for acute myocardial infarction. Two high‐sensitivity troponin I values 1 to 3 hours apart with both < upper reference level, but also > level of quantification had inadequate sensitivity and negative predictive value.
